# The Current Situation and Learning Strategies of Foreign Students in Chinese Learning Following Entrepreneurial Psychology

**DOI:** 10.3389/fpsyg.2021.746043

**Published:** 2022-01-11

**Authors:** Min Tang

**Affiliations:** School of International Education, Southwest Jiaotong University, Chengdu, China

**Keywords:** entrepreneurs’ optimistic attitude, labor law, new ventures, performance of new ventures, influence mechanism

## Abstract

Based on entrepreneurial psychology, the current situation of foreign students’ use of learning strategies in Chinese learning is explored, the overall situation of learning strategies in this process is analyzed, and the relationship between foreign students’ use of learning strategies and various factors are obtained through the designed questionnaire survey. First, a questionnaire suitable for the research respondents is designed to investigate the current situation of foreign students’ use of learning strategies in Chinese learning; second, 200 questionnaires are distributed, and 195 questionnaires are recovered, with a recovery rate of 97.5%. After the invalid questionnaire is excluded, the effective rate is 95%; furthermore, the reliability of the questionnaire data is analyzed by SPSS25 software, and Cronbach’s α coefficient is 0.869, which proves that the questionnaire has high reliability; finally, the overall situation of foreign students’ use of learning strategies in Chinese learning is analyzed from the aspects of their majors, their levels of Chinese proficiency, Chinese learning time, age and personality. The results show that the frequency of using cognitive strategies in learning Chinese is the highest, with a score of 3.689; There is a positive correlation between the use of learning strategies and the degree of proficiency of Chinese; Among them, the foreign students who have studied for 2–3 years use learning strategies the most frequently, and the students aged 28–32 use learning strategies the most frequently in the Chinese level test 4. This study provides new ideas for foreign students’ Chinese teaching and has a certain reference for foreign students’ Chinese teaching strategies.

## Introduction

As the official language of the United Nations, Chinese is well known and used all over the world. With the continuous enhancement of China’s comprehensive national strength and influence on the surrounding areas, more and more foreigners are willing to learn Chinese, and the number of foreign students is gradually increasing. But at the same time, there appear some problems. For example, the problem of Chinese learning of foreign students becomes a major obstacle to cultural exchange.

Chinese learning is one of the basic ways of human cognitive activities. Human beings acquire knowledge, improve cultural accomplishment, cultivate sentiment, and develop intelligence through Chinese learning (Jin, 2018). Krashen’s Input Hypothesis shows that enough learning materials are necessary for Chinese learning. Therefore, Chinese learning can be used as an important means to improve human ability. The quality of Chinese learning reflects the improvement of their ability (Rawendy et al., 2017). According to the information theory, the process of foreign students’ Chinese learning is the conversion process of encoding, feedback, and decoding of words, phrases, and sentences, and the encoding of speech is “comprehensible input” (Xie et al., 2019). To achieve a little higher than the existing level of Chinese learning, learners need to use some methods, which are called “learning strategies.” The so-called learning strategy is that learners make plans about the learning process purposefully and consciously to improve the learning effect and efficiency. Compared with the traditional learning methods, learning strategies can help learners accomplish learning tasks actively, and make the learning process more targeted, and the learning plan more reasonable.

Whether learners can master the learning strategies and whether they can flexibly use the mastered learning strategies largely indicate their levels of language proficiency. Therefore, the use of learning strategies is a key link in Chinese learning (Graves et al., 2018). With the rapid development of science and technology, Chinese teaching is challenged by the speed of knowledge updating. The classroom teaching focusing on basic knowledge is difficult to solve the problems faced by foreign students in their actual study (Drysdale and McBeath, 2018). Therefore, teaching foreign students to skillfully use learning strategies is an effective teaching method to cultivate their autonomous reading and learning ability, which is conducive to the development of teacher-led and learner-centered second language teaching mode (Peng et al., 2017).

As an indispensable branch of psychology, entrepreneurial psychology becomes the theoretical basis in the discipline system of Chinese international education (Fadzil et al., 2019). The research on the disciplinary system in Western countries is earlier than that in Eastern countries, and the scholars in Western countries pay more attention to the construction of the disciplinary system than those in Eastern countries. Canadian scholar Stern summed up the basic knowledge system of entrepreneurship from the 1970s to the 1980s, and incorporated entrepreneurial psychology and educational theories into the discipline infrastructure (Obschonka et al., 2020). Mr. Lü Bisong (Jena, 2020) put forward the disciplinary system in the field of international Chinese education. Luca (2017) put entrepreneurial psychology as a branch of pedagogy into the theoretical basis of the discipline system of teaching Chinese as a foreign language (Luca, 2017). Liu P. (2019) proposed to apply the principles of entrepreneurial psychology to the analysis of the Chinese language learning process.

Entrepreneurial Psychology—Entrepreneurship refers to the special ability of people to organize land, labor, capital, and other resources to produce goods, find new business opportunities, and develop new business models. Bill Gates is an entrepreneur with entrepreneurial talent. Through his excellent leadership and organizational skills, he turns Microsoft into one of the most powerful and promising companies in the world.

Zheng (2017) discussed the significance of big data and data mining in the theoretical research, practical research, and discipline construction of Chinese teaching in response to the specific problems of Chinese teaching. Combined with specific examples of Chinese teaching, the common methods and specific steps of applying data mining in Chinese teaching are discussed (Zheng, 2017). Liu P. (2019) emphasized the attention and research on online teaching and believed that the research on online learning strategies would contribute to the design of online teaching, textbook compilation, courseware production, resource construction, and learning strategy training (Liu L., 2019). Xiong (2019) believed that meeting the personalized needs of Chinese learners is a concrete manifestation of Chinese learning, the improvement of the quality of Chinese teaching, and the construction of students’ learning model through evaluation (Xiong, 2019).

Since 2007, the Office of the Academic Degrees Committee of the State Council approved that 24 universities, like Peking University, set up the Master’s degree of International Education in Chinese. There are more than 100 training institutions for the Master of International Education in Chinese in China. In the Master of Chinese International Education, curriculum construction is the core of improving teaching quality and deepening teaching reform. The research and practice of curriculum construction undoubtedly play an important role in promoting the teaching quality of the curriculum. In addition, entrepreneurial psychology is the theoretical support in the discipline system of Chinese international education. Therefore, the teaching status and strategies of Chinese language learning are discussed from the perspective of entrepreneurial psychology to promote Chinese language learning.

The first chapter introduces the relevant theories and research results of Chinese language learning in China and foreign countries; the second chapter describes the purpose, research object, and the design of the survey first, and then the survey data are processed; the third chapter analyzes the learning strategies of foreign students’ Chinese language according to the data from the relationship between learning strategies and majors, the relationship between learning strategies and Chinese proficiency, and the relationship between learning strategies and age, as well as personality. It is found that there are still some problems in foreign students’ Chinese education, and finally, some corresponding suggestions are given. The fourth chapter draws the corresponding conclusion according to the research results.

The current situation of the use of learning strategies in Chinese learning of foreign students in a key university in Beijing is investigated. Under the guidance of entrepreneurial psychology, the correlation between teaching strategies is studied. The important significance for teaching and learning in Chinese learning in foreign students’ education is discussed from the aspects of major, Chinese proficiency, age, and personality. On this basis, the significance of Chinese teaching and learning for foreign students is discussed. The innovation of this study is to combine the concept of entrepreneurial psychology with Chinese learning strategies of foreign students, which provides a reference for Chinese teaching for foreign students.

## Research Method and Content

### Entrepreneurial Psychology—Entrepreneurship

The concept of “entrepreneur” is first proposed by the French economist Richard Cantillon in 1800. That is, entrepreneurs, turn the efficiency of economic resources from low to high; “Entrepreneurship” a collection of entrepreneurs’ special skills (including spirit and skills). In other words, “entrepreneurship” refers to the expression of the comprehensive ability of entrepreneurs to organize, establish and manage enterprises. It is an important and special intangible factor of production. For example, the greatest “product” created by the great entrepreneurs and founders of Sony Corporation, Akio Morita and Kenda Inoue, is not a tape recorder or a bar color picture tube but Sony Corporation and everything it represents; Walt Disney’s greatest creation is not puppet adventures, nor snow-white, nor even Disneyland, but Walt Disney Company and its extraordinary ability to make the audience happy; Sam Walton’s greatest creation is not “constant daily parity” but Wal Mart, an organization that can turn retail essentials into action in the best way. In the nineteenth century, people summarize some characteristics of entrepreneurs as entrepreneurship. In the use of English terms, entrepreneurs and entrepreneurship are often interchanged. For a long time, the concept of an entrepreneur is usually defined from the aspects of business, management, and personal characteristics. After the twentieth century, the abstract concept of entrepreneur—the definition of entrepreneurship is extended to the fields of behavior, psychology, and sociological analysis. In the developed countries, it is very common for entrepreneurs to work in government or social organizations. They also constantly propose and implement the use of entrepreneurship to transform government service and social management. Nowadays, China’s entrepreneurship can be summarized as follows: (a) Developing spirits: Improve the business environment and return entrepreneurs equality; Clarify the government and market boundaries and expand the growth space of entrepreneurship; Build a clean and pro-government business relationship and endow entrepreneurs with spiritual justice; Protect entrepreneurs’ property rights and enterprise intellectual property rights according to laws; Create a good social and cultural ecology and cultivate the soil of entrepreneurship. (b) Innovative spirits: The essence of innovation is to create new value for customers, not technology and concept itself. (c) Opportunity vigilance and sensitivity: Entrepreneurship must be alert and sensitive to opportunities, correctly distinguish what opportunities are and what pseudo opportunities are, and the time node cannot be too early or too late. According to the five elements of success, opportunities efficiently should be turned into value. (d) Take risks and uncertainties: turning uncertainty into certainty can minimize risks. (e) Grit: It is the sustained passion and endurance for long-term goals. It is not forgetting the original intention, concentration, investment, and perseverance. It is a personality feature that includes self-motivation, self-discipline, and self-adjustment. (f) Continuous and effective learning: deconstruct self-cognition, change thinking modes, examine the ideas behind actions, reflect on our mental model, thinking logic, and one-sidedness of knowledge and information, and then correctly and effectively adjust our mental model, thinking logic, or expand knowledge and information in new fields. The ability is improved by adhering to effective learning. (g) Craftsman spirits: Innovative spirit and craftsman spirit are the core competitiveness. At present, China is following the trend and has made gratifying achievements in implementing the innovation of curve overtaking in the Internet and artificial intelligence. However, the innovative spirit and craftsmanship spirit are still at a disadvantage in the global competition in traditional industries because the industrialization time is short. H. value-oriented principles: Value orientation is mostly transformed from poverty and opportunity-driven; if self-motivation is continuous, sustained success will be achieved. What kind of value orientation an organization has means what kind of future it has. Value orientation is in line with entrepreneurship.

### Purpose of the Survey

The purpose of the survey is to study the state and teaching status of foreign students in Chinese language learning, as well as the improvement methods of teaching strategies. According to entrepreneurial psychology, the current situation of Chinese learning strategies of foreign students is investigated. Through the data analysis of the survey, the suggestions to improve the quality of Chinese teaching are put forward to help students to arouse their interest in Chinese learning (Liu and Gao, 2020) and improve their ability to learn Chinese. In this study, the research questions mainly fall into five parts: The overall situation of foreign students’ use of learning strategies in Chinese learning, the relationship between foreign students’ use of learning strategies and their majors, Chinese proficiency, ages, and personalities. The following is a brief introduction to the survey content.

### Investigation Content

The content of this study mainly starts from five points: The overall state of the use of learning strategies by foreign students in Chinese learning, the relationship between the use of learning strategies by foreign students in Chinese learning and whether they are Chinese related majors, the relationship between the use of learning strategies by oversea students in Chinese learning and their Chinese proficiency, the relationship between the use of learning strategies by oversea students in Chinese learning and the age of oversea students, the relationship between the use of learning strategies and the personality of oversea students in Chinese learning. Next, the research subjects are selected.

### Research Subjects

Foreign students in a key University in Beijing are taken as the research object, and the number of foreign students in the university is 354. In this study, 200 students are randomly selected to distribute questionnaires, including 115 students majoring in Chinese and 85 students majoring in non-Chinese. The foreign students in the elementary Chinese class, intermediate Chinese class, advanced Chinese class, and Master of Chinese Education are listed in the ranks of Chinese majors in this study, while those in the Pakistan Medical Class and Doctoral programs of different ages are listed in the ranks of non-Chinese-related majors. The design of the survey and questionnaire is described as follows.

### Survey Design

The questionnaire survey is used to collect data, and the design of the questionnaire mainly refers to the scale of the survey of reading strategies and the scales designed by predecessors (Baskin et al., 2017). After the actual situation of the schools where the objects in this study are located is analyzed, the scale of learning strategies used by the foreign students in Chinese learning is designed. The scale used in this study is mainly from three angles (Hederich-Martínez et al., 2018): (1) Basic information of the foreign students. It includes their nationality, major, Chinese learning duration, levels of Chinese proficiency, genders, ages, and personalities; (2) the current situation of the foreign students’ use of the learning strategies in Chinese learning. Although the content in this part is general, it helps make foreign students have the concept of learning strategies; (3) the investigation of each specific learning strategy contained in the four types of learning strategies. The content in this part is detailed and comprehensive, which can deeply study the use of learning strategies when foreign students study Chinese, and then the relations between the use of learning strategies and their majors, levels of Chinese proficiency, ages, and personalities are discussed. According to the survey results, corresponding suggestions are put forward. Previous studies focus on the study of foreign students’ learning behavior, which is divided into direct strategies and indirect strategies, and students’ learning strategies are analyzed from the aspects of psychology, emotion, cognition, reversal, memory, and compensation.

**FIGURE 1:**
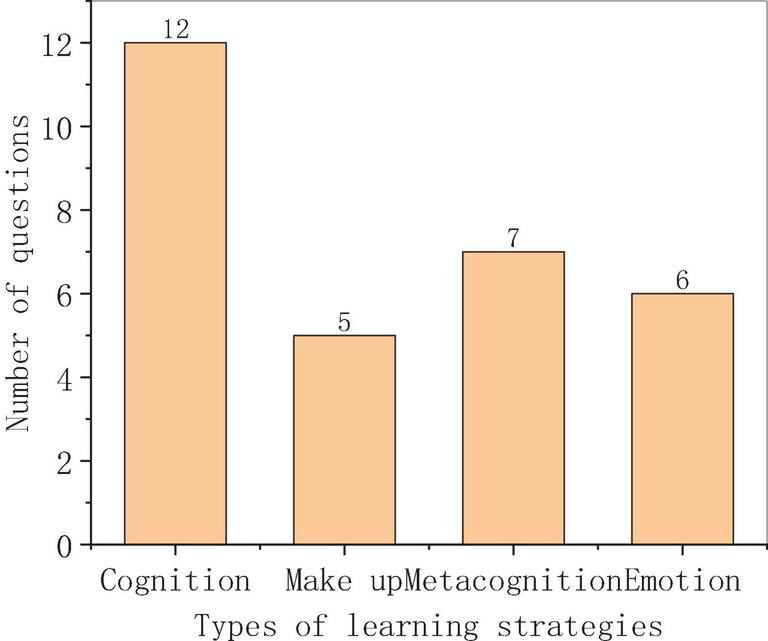
Number of learning strategies.

Oxford points out that the concept of learning strategies is the learning behavior adopted by learners for a more successful, autonomous, and interesting study of Chinese (Alliprandini et al., 2017). The concept of learning strategies defined by Alice is used, that is, learners’ psychological behavior and action are associated in a particular stage, or the learning process, or the use of knowledge (Tran et al., 2019). Oxford divides learning strategies into direct strategies and indirect strategies. Direct strategies include memory, make-up, and cognition, and indirect strategies are affective, social, and metacognitive strategies. In this study, four types of learning strategies are selected, including cognition, make-up, metacognition, and emotional strategies (Ulstad et al., 2018). In the questionnaire, 1–11 questions are the content about the cognitive strategy, 12–17 questions are about the make-up strategy, 18–23 are about the metacognitive strategy, and 24–0 are about the emotional strategy. The total number of questions in each type is 12, 5, 7, and 6. In addition, these four types of learning strategies are specifically classified according to the classification criteria. After Oxford, Mokhtari, and Sheorey’s scales of learning strategy are referred to, the items in the questionnaire are set according to the relevant contents of this study, increasing the diversity of the questionnaire items, making the study more targeted and objective (García-Pérez et al.). The Likert scale is introduced, that is, each item is set 1–5 options, corresponding to five levels of “disagree totally,” “disagree,” “uncertain,” “agree” and “agree.” The data are collected and analyzed by using SPSS25 (Han et al., 2018). The number of each learning strategy is set as shown in Figure 1.

After the questionnaire data are obtained, the data needs to be sorted, processed, and analyzed. The steps and tools of data analysis are as follows.

### Data Processing

A total of 200 questionnaires are distributed and 195 are recovered, which includes 113 Chinese majors and 82 non-Chinese majors, with a recovery rate of 97.5%. Five invalid questionnaires are excluded, and 190 valid questionnaires are finally obtained, with an effective recovery rate of 95%. This shows that the questionnaire is feasible. In addition, the study uses Cronbach’s α coefficient for reliability analysis of the questionnaire (McNeish, 2018). In general, if the reliability coefficient is less than 0.6, it is considered to be insufficient. The reliability coefficient between 0.7 and 0.8 is basically credible, and the reliability coefficient between 0.8 and 0.9 is very credible (Jain and Angural, 2017). After the reverse comparison items are excluded, the reliability analysis is carried out by SPSS25 software, and the reliability coefficient is 0.869, which proves that the questionnaire in this study has high reliability (Vanus et al., 2019).

## Analysis of the Survey Results of the Use of Learning Strategies of Foreign Students’ Chinese Learning

### Analysis of the Overall Situation of the Use of Learning Strategies of Foreign Students’ Chinese Learning

**FIGURE 2:**
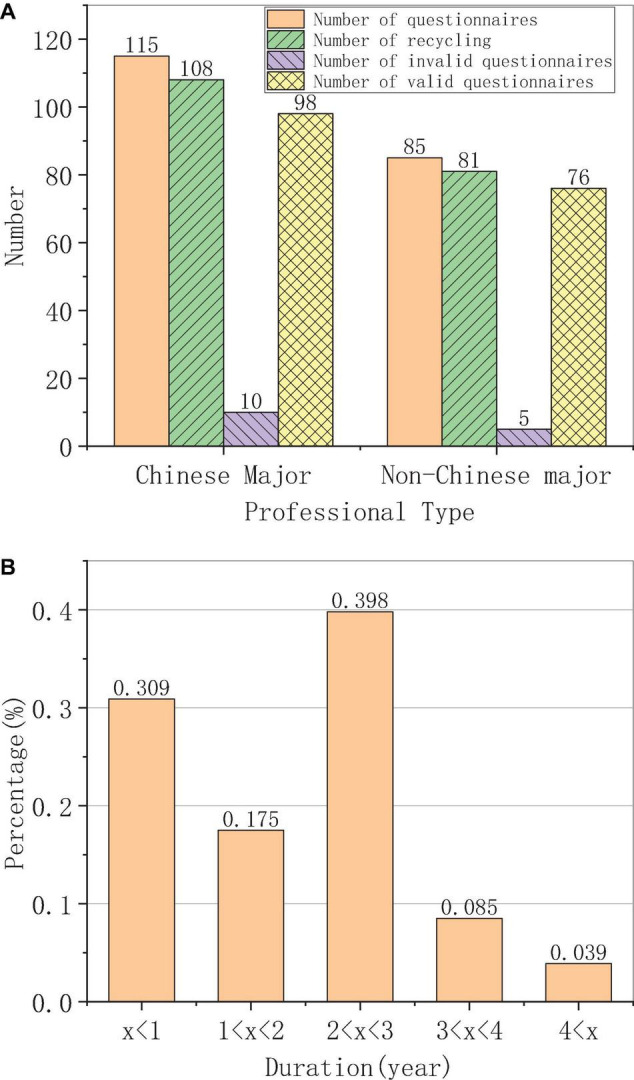
Professional type and Chinese learning duration of foreign students. **(A)** Professional distribution. **(B)** Chinese learning duration.

The analysis of the survey results of foreign students’ Chinese learning duration is shown in Figure 2.

The major distribution of the questionnaire is shown in Figure 2A. The distribution of foreign students’ Chinese learning is shown in Figure 2B.

Figure 2A shows that a total of 200 questionnaires are distributed, and the data of 113 Chinese majors and 82 non-Chinese majors are collected, with a recovery rate of 97.5%. Five invalid questionnaires are excluded, and 190 are valid and used for analysis. The results in Figure 2B show that there are a large number of foreign students majoring in Chinese, and the final recovery rate and the effective recovery rate are slightly lower than those of non-Chinese majors, accounting for about 40% of the total, followed by the number of foreign students who study Chinese less than 1 year, accounting for about 31%. The proportion of the students who study Chinese for 1–2 years is about 17%, and the number of the students who study Chinese for 3–4 years and more than 4 years does not reach 10% of the total number. This shows that the number of students who insist on learning Chinese is still small. This phenomenon is affected by many factors. For example, Chinese is difficult, China’s position in the international community is low, and foreign students feel that learning Chinese is not necessary.

**FIGURE 3:**
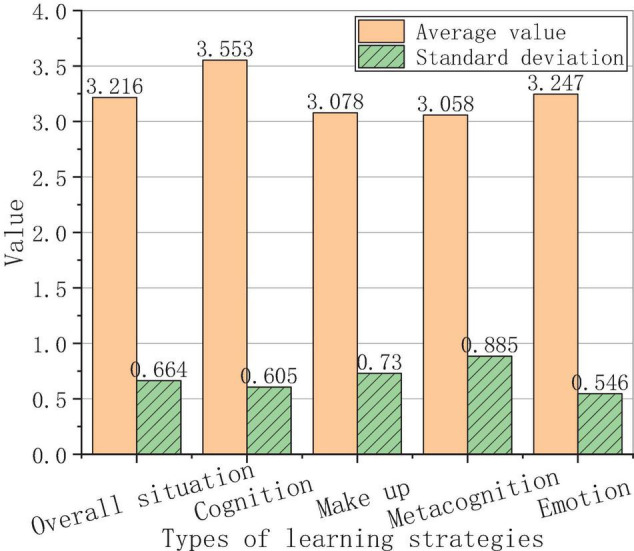
Analysis of the use of each learning strategy.

The analysis of the average score and the standard deviation of the use of each learning strategy is shown in Figure 3.

Figure 3 shows that the learning strategies are divided into cognitive strategies, make-up strategies, metacognitive strategies, and emotional strategies. The average score of the overall situation of the four types of learning strategies is 3.165, which is an upper-middle value, indicating that the students in this study have a strong consciousness of using learning strategies. Specifically, foreign students have the highest frequency of using cognitive strategies, with an average score of 3.548, followed by emotional strategies, with an average score of 3.324. The average scores of the make-up strategies and metacognitive strategies are 3.032 and 3.011, respectively. In terms of standard deviation, the biggest one is the metacognitive strategy, which reaches 0.852; the second is the compensation strategy, which reaches 0.756; then there is cognitive strategy, reaching 0.661; finally, the affective strategy reached 0.539; the overall average value is 0.702. Obviously, foreign students score the lowest in the metacognitive strategies, indicating that foreign students’ ability of self-control is poor, and they are not autonomous in Chinese learning.

**FIGURE 4:**
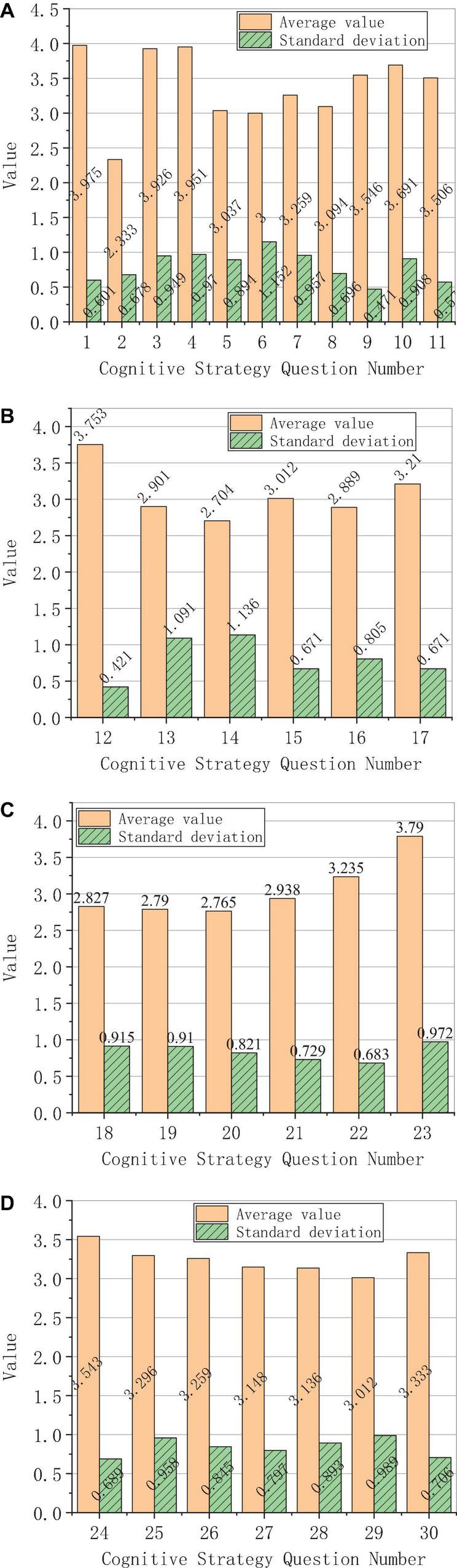
Results of the specific use of learning strategies. **(A)** Cognitive strategies. **(B)** Make-up strategies. **(C)** Metacognitive strategies. **(D)** Emotional strategies.

The analysis of the survey results of the specific use of each learning strategy is shown in Figure 4.

The results of cognitive strategies are shown in Figure 4A, the results of compensation strategies are shown in Figure 4B, the results of metacognitive strategies are shown in Figure 4C, and the results of emotional strategies are shown in Figure 4D.

Figures 4A–D, respectively, respond to the cognitive strategies, the make-up strategies, the metacognitive strategies, and the emotional strategies. In the cognitive strategies, the learning strategy, which is the least used, is induction, and the corresponding question number is 2, with an average score of only 2.294. The most frequently used learning strategy is translation, the corresponding question number is 1, with an average value of 3.957. In general, the trend of the use frequency of each specific learning strategy in cognitive strategies is polarized, and the use frequency of make-up strategies and metacognitive strategies is relatively equal, and the use of emotional strategies is higher. This proves that foreign students pay more attention to “translation,” ignoring “induction and conclusion” in the process of Chinese learning.

**FIGURE 5:**
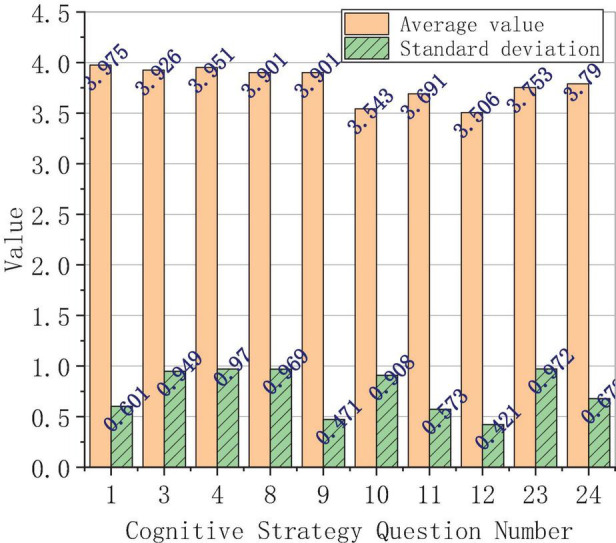
Analysis of the top 10 types of learning strategies used in Chinese learning.

The analysis of the top 10 types of learning strategies used is shown in Figure 5.

Figure 5 shows the 10 learning strategies that are most frequently used in Chinese learning. The 10 analysis strategies in the figure are the translation strategy, the reading seeking strategy, the skipping strategy, the analysis strategy, the word guessing strategy, the evaluation strategy, the squeaking cognitive strategy, the self-monitoring strategy, the anxiety reduction strategy, and the summary strategy. The strategies in the figure are classified by the number of titles. Cognitive strategies account for the highest, and there is only one in make-up strategies, metacognitive strategies, and emotional strategies, respectively. Except for the translation strategy in cognitive strategies, foreign students also use the reading-seeking strategy most frequently, with an average score of 3.961, followed by skipping, with an average score of 3.689. Other frequently used strategies include analysis, guessing, and evaluation. In general, foreign students’ ability of cognition and self-monitoring is not strong, but they are skilled in using knowledge, which shows that foreign students have good knowledge of their levels of Chinese proficiency. In addition, it has to be noted that among the emotional strategies, foreign students have a higher score in reducing anxiety, with an average score of 3.545, indicating that foreign students use this strategy more frequently, and almost everyone can alleviate their anxiety and pressure in Chinese learning.

### Analysis of the Survey Results of the Relations Between the Use of Learning Strategies and Their Majors

**FIGURE 6:**
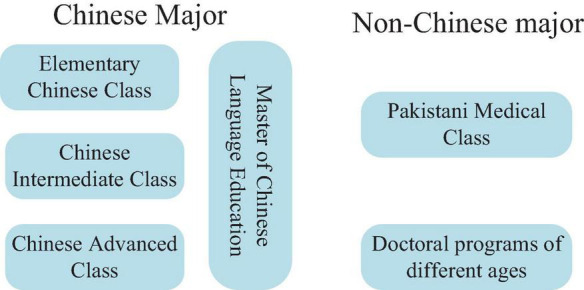
Analysis of the survey results of the relations between learning strategies and their majors of foreign students.

The major distribution of foreign students is shown in Figure 6.

The survey results in Figure 6 show that the research objects can be divided into two categories according to their majors: Chinese majors and non-Chinese majors. Among them, Chinese majors include Master of Chinese language Education, elementary Chinese class, Chinese intermediate class, and Chinese advanced class. The non-Chinese majors include Pakistan Medical Class and Doctoral programs of different ages. The survey on Chinese learning strategies of foreign students from different majors can reflect the comprehensiveness of the results of this study.

**FIGURE 7:**
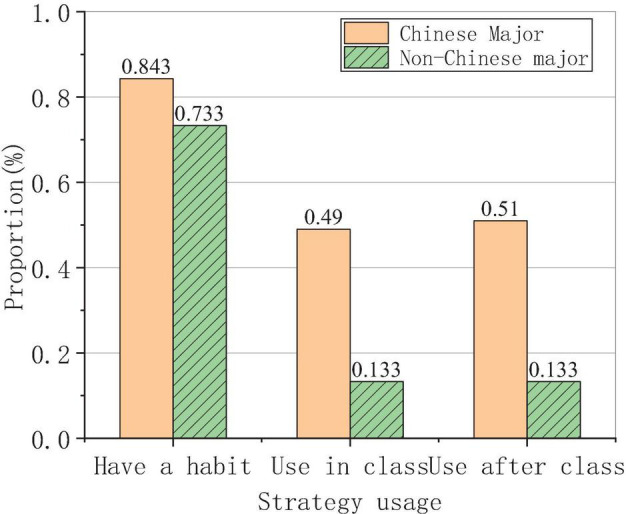
The use of learning strategies for Chinese majors and non-Chinese majors.

The use of learning strategies for Chinese majors and non-Chinese majors is shown in Figure 7.

The survey results in Figure 7 show that the proportion of the students majoring in Chinese who use learning strategies in Chinese learning accounts for more than 80%, while that of non-Chinese students accounts for about 72%. The percentage of the students majoring in Chinese who can use learning strategies in the classroom is about 50%, while that of the students majoring in non-Chinese is only about 10%. The use of learning strategies after class between the two is similar. Students majoring in Chinese have stronger consciousness of Chinese learning than students majoring in non-Chinese.

**FIGURE 8:**
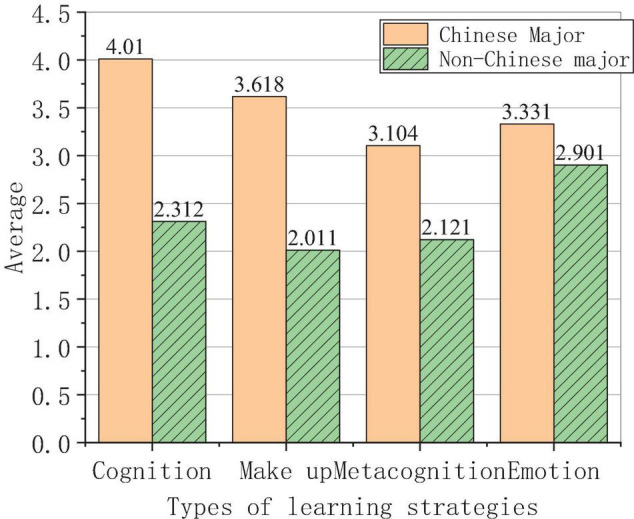
Use of specific learning strategies for different majors.

The specific use of the learning strategies of different majors is shown in Figure 8.

The survey results in Figure 8 show that the average scores of Chinese majors are more than 3, while the average scores of non-Chinese majors are about 2, indicating that the use of learning strategies among Chinese majors is significantly higher than that among non-Chinese majors, which further confirms that Chinese majors have a higher consciousness of learning Chinese than those of non-Chinese majors. The cognitive strategy is the most frequently used learning strategy among Chinese majors, and emotional strategies are the most frequently used learning strategy among non-Chinese majors, which is consistent with the characteristics of their major.

### Analysis of the Relation Between the Use of Learning Strategies and Their Levels of Chinese Proficiency

**FIGURE 9:**
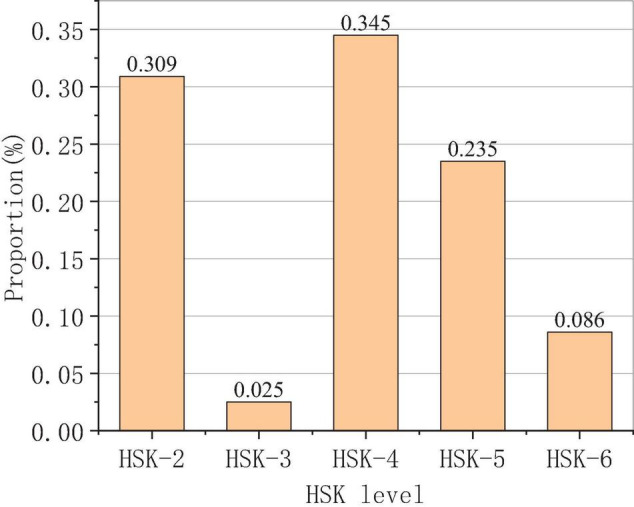
Results of foreign students’ HSK level.

The results of foreign students’ HSK (Hanyu Shuiping Kaoshi) are shown in Figure 9.

The survey results in Figure 9 show that the students in this study have passed the HSK (Chinese proficiency test), of which about 30% have passed HSK-2, 35% have passed HSK-4, 23% have passed HSK-5, and the rest have passed HSK-3 and HSK-6, respectively. The levels of HSK are divided into the following degrees for analysis: HSK-2 and HSK-3 are primary levels, HSK-4 is the middle level, and HSK-5 and HSK-6 are high levels. The number of foreign students in each level accounts for about 30%, indicating that the survey results are more objective.

**FIGURE 10:**
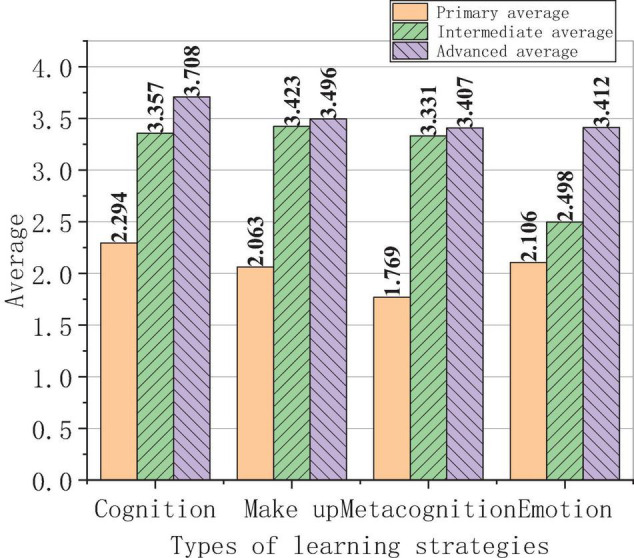
Relation between the use of learning strategies of foreign students and their different levels of Chinese proficiency.

The relation between the use of learning strategies of foreign students and their different levels of Chinese proficiency is shown in Figure 10.

The survey results in Figure 10 show that there is a significant gap in the use of learning strategies among students of different HSK levels. Primary students rarely use cognitive strategies, accounting for 2.294; those using compensation strategies account for 2.063, the number of the students using metacognitive strategies accounts for 1.769, and the students who use emotional strategies account for 2.106. The proportion of the students using learning strategies is still very low. Intermediate students using cognitive strategies account for 3.357, using compensation strategies account for 3.423, using metacognitive strategies account for 3.331, and using emotional strategies account for 2.498. The proportion of the intermediate students is higher than that of primary students. Intermediate and senior students using cognitive strategies account for 3.708, using compensation strategies account for 3.496, using metacognitive strategies account for 3.407, and using emotional strategies account for 3.412. The proportion is the highest among all the students. The use frequency of learning strategies used by primary and middle-level students is generally low. The use frequency of learning strategies used by middle-level students increases slightly, and their use frequency of emotional strategies is still low. The use frequency of learning strategies used by middle and high-level students is above average, and their use frequency of cognitive strategies is the highest. The average scores of cognitive strategies among primary, middle, and high-level students are 2.501, 3.362, and 3.710, respectively. The average scores of make-up strategies are 2.065, 3.451, and 3.952, respectively. The average scores of metacognitive strategies are 1.784, 3.351, and 3.452, respectively. The average scores of emotional strategies are 2.105, 2.531, and 3.414. According to the data, it is concluded that the higher the HSK level of foreign students is, the more skilled they use learning strategies is. With the increase of their levels of Chinese proficiency, the more flexible they use make-up strategies. This illustrates the role of learning strategies in improving foreign students’ levels of Chinese proficiency. The compensation strategy plays a strong role in Chinese language learning, and the compensation strategy promotes the construction of students’ cognitive ability and self-regulation ability. And their self-monitoring ability and cognitive ability will be gradually improved. In general, the HSK level of students is positively correlated with their use of learning strategies, and the higher the level is, the more frequently the students use learning strategies.

### Analysis of the Relation Between the Use of Learning Strategies of Foreign Students and Their Ages and Personalities

**FIGURE 11:**
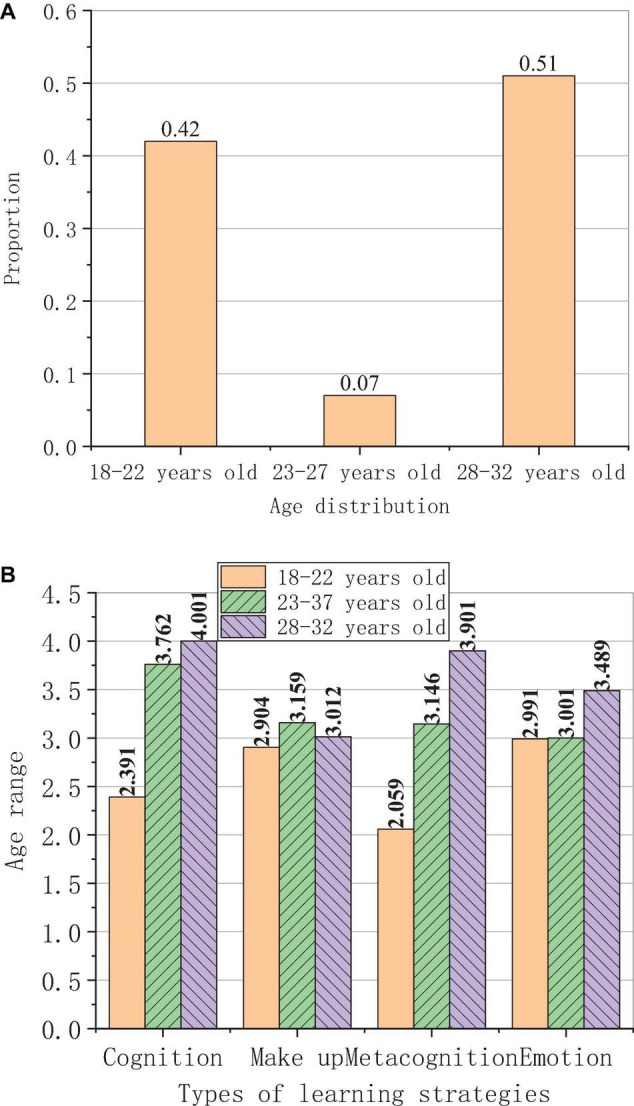
Survey results of the relations between learning strategies used by foreign students and their ages. **(A)** Ages. **(B)** Use of different Chinese learning strategies.

The relationship between age and the use of learning strategies of foreign students as shown in Figure 11.

The age distribution of the respondents is shown in Figure 11A, and the use of strategies at different ages is shown in Figure 11B.

The survey results in Figure 11A show that the ages of the foreign students in this study are divided into 18–22 years old, 23–27 years old, and 28–32 years old. Among them, 23–27 years old is the most, accounting for about 55%, followed by 18–22 years old, accounting for about 40%, and the rest is 28–32 years old. This shows that the age of most people who learn Chinese is under 27 years old. This is because people are unwilling to learn new things as they grow older. Figure 11B shows that the older the student is, the higher the use frequency of learning strategies is. The average scores of the use frequency of cognitive strategies in the three age groups are 2.395, 3.763; and 4.002; the average scores of make-up strategies are 2.905, 3.156, and 3.021; the average scores of metacognitive strategies are 2.061, 3.151, and 3.905; the average scores of the emotional strategies are 2.995, 3.021, and 3.453. This shows that the increase of age has an obvious effect on the use of learning strategies. For cognitive strategies, the improvement effect is the most obvious at the age of 18–27, reaching 1.368, and it only increases by 0.239 at the age of 23–32, which shows that cognitive learning strategies promote learning ability most in youth; For affective learning strategies, the improvement effect is not obvious at the age of 18–27, and it increases by 0.026 and increases by 0.432 at the age of 23–32, which shows that affective learning strategies promote learning ability most at older ages; For the compensation strategy, the improvement effect is the most obvious at the age of 18–27, reaching 0.251, while it decreases by 0.135 at the age of 23–32, which shows that the affective learning strategies promote the learning ability most at younger ages, but hinders the improvement of learning ability at older ages; For metacognitive strategies, the improvement effect is the most obvious at the age of 18–27, reaching 1.088, and it increases by 0.749 at the age of 23–32, which shows that affective learning strategies promote learning ability the most when they grow older.

**FIGURE 12:**
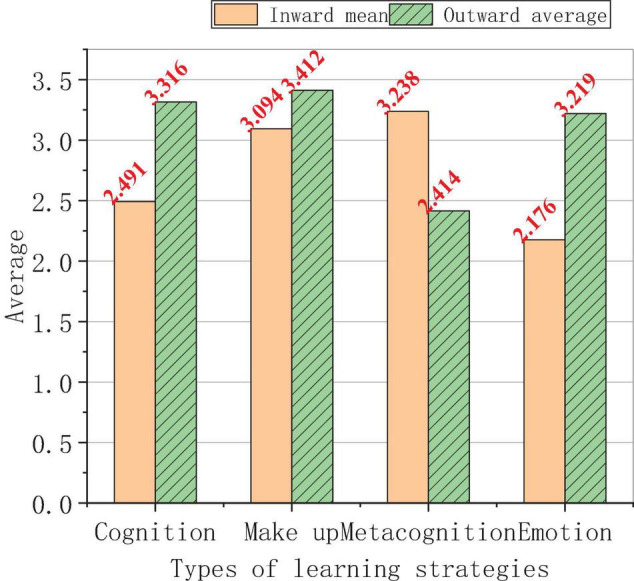
Relation between the students’ personalities and their use frequency of learning strategies.

The analysis of the relation between the student’s personalities and their use frequency of learning strategies is shown in Figure 12.

Figure 12 shows that the students with introverted and extroverted personalities use metacognitive strategies the most frequently, but their use frequency of the emotional strategies is the lowest. Overall, the average score of the extroverted students is higher than that of the introverted students, that is, extroverted students use learning strategies more frequently than introverted students. This also verifies that the characteristics of cognitive strategies are more suitable for students with active thoughts. There is little difference in the use frequency of make-up strategies between introverted and extroverted foreign students. The introverted students in the use frequency of metacognitive strategies are higher than the extroverted students. The reason may be that the introverted students are better at in-depth thinking, and there is a large gap in the use frequency of emotional strategies between the introverted and the extroverted students. This shows that introverted students are more likely to have psychological burdens, often unable to find suitable methods of releasing their pressure, and more likely to suffer from depression. However, extroverted students are more willing to express and share their ideas and they have many ways to deal with their negative emotions.

### Suggestions on the Use of Learning Strategies for Foreign Students

According to the analysis of the survey result, it can be found that there are still common problems in the current Chinese education for foreign students. For example, although the overall frequency of foreign students’ use of learning strategies in Chinese learning is more than half, the scores of foreign students in the classification of metacognitive strategies are low, indicating that foreign students have plans in Chinese learning, but their self-monitoring ability is weak. Other problems existing in Chinese learning have been analyzed above, and the corresponding measures should be taken to improve the effectiveness of the level of Chinese proficiency of foreign students (Guo and Leung, 2021).

First, in terms of the foreign students’ Chinese education, teachers should guide them to develop their learning habits in Chinese teaching activities: (1) teachers can guide them by asking foreign students to quickly browse Chinese textbooks and skimming skills in the classroom, or by deliberately practicing summary and summarizing articles to improve their learning ability (Gao and Shen, 2021); (2) since foreign students are highly dependent on translating articles in Chinese learning, teachers can alleviate the phenomenon by reducing the time of reading Chinese textbooks in the classroom. If foreign students want to complete the understanding of textbooks in a limited time, they must cut the time used for translation (Weerasawainon, 2019); (3) it is necessary to post appropriate learning pressure to improve the learning efficiency of foreign students in Chinese learning. According to the survey results, foreign students have a high frequency of using the learning strategy of reducing anxiety in the examination, so students have the corresponding ability to reduce anxiety. In general, it is necessary to appropriately increase their learning pressure and create a slightly tense classroom environment to help foreign students use the learning strategy (Zhou, 2020). When the frequency of using this strategy increases, the learning efficiency of foreign students will improve (Bruen, 2020); (4) teachers should often remind themselves that if you give a man a fish, you have fed him for today; and if you teach a man to fish, you have fed him for a lifetime. It is important to educate students to use learning strategies. And more importantly, teaching students to use learning strategies independently can make them develop a good habit of autonomous learning and helps students improve their learning efficiency fundamentally (Li et al., 2018).

Second, teachers should teach students in accordance with their aptitude, and try to achieve this goal through some strategies (Collie et al., 2017): (1) for the students from non-Chinese majors, some interest classes in Chinese learning can be opened following the school system, and the learning strategies that can be applied in Chinese learning in interest classes, and targeted special training can be conducted (Yang, 2020); (2) for the foreign students with different levels of Chinese proficiency, teachers should emphasize their basic knowledge, and require students to pay attention to the accumulation of knowledge in learning; (3) the awareness of using metacognitive strategies should be cultivated from the initial stage of their Chinese learning. Only by cultivating independent learning habits according to their learning characteristics can other learning strategies be flexibly used (Huang, 2017); (4) in view of the difference in the use frequency of teaching strategies caused by different ages, teachers should pay attention to the psychological impact on the learning of foreign students aged 18–22, and strengthen situational teaching methods to help the students better use emotional strategies, alleviating their anxiety caused by learning pressure and improving their learning efficiency (Lin et al., 2017); (5) for the students with different personalities, teachers should pay more attention to the cultivation of learning strategies used by the students with introverted personalities. Their scores in cognitive strategies, make-up strategies, and emotional strategies are relatively low, especially in the use of emotional strategies. Improving the use of the strategy can help foreign students with introverted personalities improve their ability to regulate their psychological emotions, eliminating their negative emotions, and finally achieving the effect of Chinese learning (Yao, 2017). Foreign students with extroverted personalities also need to pay attention to guide them to use metacognitive strategies. Only by improving the ability of autonomous learning can the use of other learning strategies be improved (Wang, 2017).

The nine suggestions in the above are proposed based on this investigation and study, which may be more suitable for the research object and more suitable for their learning situation. And they also reflect the general characteristics of some foreign students in Chinese learning. It is hoped that the study can have significance to the Chinese teaching of foreign students in other colleges and universities.

## Conclusion

Based on entrepreneurial psychology, the current situation of the use of learning strategies by foreign students in Chinese learning is investigated, and the relations between learning strategies used by foreign students in Chinese learning and their majors, levels of Chinese proficiency, Chinese learning duration, ages, and personalities are explored. First, a questionnaire suitable for the research objects is designed according to the questionnaire related to learning strategies, and then 200 questionnaires are distributed and 195 are recovered, with a recovery rate of 97.5%. After invalid questionnaires are excluded, the effective rate is 95%. Finally, the reliability of the questionnaire data is analyzed by SPSS25, and the Cronbach’s α coefficient is 0.869, which proved that the questionnaire has high reliability. The survey results show that in the overall use of learning strategies, foreign students have the highest frequency of using cognitive strategies in the process of Chinese learning. The use of learning strategies in Chinese learning is positively correlated with their majors. Foreign students who have studied Chinese for 2–3 years most frequently use learning strategies in Chinese learning. Foreign students with HSK-4 most frequently use learning strategies in Chinese learning. Foreign students aged between 28 and 32 have the highest frequency of using learning strategies. The above survey and analysis results are relatively comprehensive, but there are still some shortcomings. Therefore, it is necessary to emphasize the use of Chinese learning strategies in teaching, so that students can quickly improve their levels of Chinese proficiency, and use entrepreneurial psychology to increase foreign students’ enthusiasm for Chinese learning. For example, the nationality of the research objects is relatively single, and the suggestions proposed for foreign students in Chinese learning have not yet been verified, which will be the direction of the next research in the future.

## Data Availability Statement

The raw data supporting the conclusions of this article will be made available by the authors, without undue reservation.

## Ethics Statement

The studies involving human participants were reviewed and approved by the Southwest Jiaotong University Ethics Committee. The patients/participants provided their written informed consent to participate in this study. Written informed consent was obtained from the individual(s) for the publication of any potentially identifiable images or data included in this article.

## Author Contributions

The author confirms being the sole contributor of this work and has approved it for publication. MT proposed the research topics, designed the research schemes, researched and organized the literature, drafted the documents, collected and sorted out data, and completed the manuscript.

## Conflict of Interest

The author declares that the research was conducted in the absence of any commercial or financial relationships that could be construed as a potential conflict of interest.

## Publisher’s Note

All claims expressed in this article are solely those of the authors and do not necessarily represent those of their affiliated organizations, or those of the publisher, the editors and the reviewers. Any product that may be evaluated in this article, or claim that may be made by its manufacturer, is not guaranteed or endorsed by the publisher.
